# Great Men Differ

**Published:** 1895-01

**Authors:** 


GREAT MEN WILL DIFFER.—It is well known that the American Medical Association favors a Bureau of Public Health with a health commissioner in the cabinet.—It is also well known that the New York Academy of Medicine are opposed to such movement, and favor a National Board of Health. It is strange how men look at the same thing through different glasses. The President, in his message to Congress, favored legislation in the interest of the public health, and here is what he said: let our readers construe it and decide which is right, the Academy of Medicine organ—the New York *Medical Record*, or the American Medical Association's organ—their journal:

President Cleveland, in his message to Congress, makes the following recommendation: "I am entirely convinced that we ought not to be longer without a National Board of Health or national health officer, charged with no other duties than such as pertain to the protection of our country from the invasion of pestilence and disease. This would involve the establishment, by such board or officer, of proper quarantine precautions, or the necessary aid and counsel to local authorities on the subject, prompt advice and assistance to local boards of health or health officers in the suppression of contagious disease, and in cases where there are no such local boards or officers, the immediate direction by the National Board or officer of measures of suppression, constant and authentic information concerning the health of foreign countries and all parts of our own country, as related to contagious diseases; and consideration of regulations to be enforced in foreign ports to prevent the introduction of contagion into our cities, and the measures which should be adopted to secure their enforcement. There seems to be at this time a decided inclination to discuss measures of protection against contagious diseases in international conference, with a view of adopting means of mutual assistance. The creation of such a national health establishment would greatly aid our standing in such conferences, and improve our opportunities to avail ourselves of their benefits. I earnestly recommend the inauguration of a National Board of Health, or similar national instrumentality, believing the same to be a needed precaution against contagious disease, and in the interest of the safety and health of our people."

What the New York *Medical Record* said: "It can be easily seen, by the wording of his recommendation, that the establishment of a new Department of Government is not intended or considered at present necessary. This is the view which has been taken by leading sanitarians all over the country, and is the one that has been voiced by the Academy of Medicine in its carefully prepared bill."

What the *Journal* A. M. A. said: "It is very apparent from the President's words, that he would lose no time in approving an act establishing a *Department of Public Health*."

